# Phototropin-mediated blue light signaling orients the asymmetry of *Marchantia polymorpha* spores

**DOI:** 10.1126/sciadv.aei3142

**Published:** 2026-11-11

**Authors:** Johannes Roetzer, Radka Slovak, Eva-Sophie Wallner, Natalie Edelbacher, Beate Asper, Sebastian Deiber, Sebastian Seitner, Martin Colombini, Liam Dolan

**Affiliations:** ^1^ Gregor Mendel Institute of Molecular Plant Biology, 1030 Vienna, Austria.; ^2^ Vienna BioCenter PhD Program, A Doctoral School of the University of Vienna and Medical University of Vienna, 1030 Vienna, Austria.; ^3^ Department of Biology, University of Oxford, South Parks Road, Oxford OX1 3EL, UK.; ^4^ Plant Sciences Facility, Vienna BioCenter Core Facilities GmbH (VBCF), 1030 Vienna, Austria.; ^5^ Mechanical Engineering Center, Institute of Molecular Pathology, 1030 Vienna, Austria.

## Abstract

Multicellular organisms produced by sexual reproduction develop from single cells, and the asymmetry of these cells can define the orientation of the earliest developmental axes. The haploid multicellular stage of the plant, *Marchantia polymorpha,* develops from a single cell—the spore—that divides asymmetrically, producing an apical germ cell that generates the plant body and a smaller basal cell that differentiates as an anchoring germ rhizoid cell. We show that the orientation of this asymmetric cell division is controlled by an external environmental cue—blue light—that is perceived by the photoreceptor phototropin. This process requires the NCH1 (NRL PROTEIN FOR CHLOROPLAST MOVEMENT 1) protein for downstream signaling. Together, these findings define core elements of the mechanism by which a directional environmental signal orients cell division, which, in turn, orients the first axis of symmetry.

## INTRODUCTION

Land plants develop separate, multicellular haploid and diploid phases in their life cycle. The diploid phase develops from a zygote produced by the fertilization of a polarized egg (*1*). The polarity established in the egg is inherited by the zygote (*2*), which orients the first cell division and aligns the apical-basal axis of the embryo through a mechanism in which auxin modulates the activity of transcription factors in different regions of the embryo (*3*). In flowering plants, the dominant phase of the life cycle is diploid, and the haploid phase is highly reduced developing within ensheathing parental tissues. By contrast, in bryophytes such as *Marchantia polymorpha*, the dominant multicellular phase is haploid, and the diploid phase is relatively small and develops embedded in haploid tissue. The haploid phase of *M. polymorpha* develops from the cells produced by meiosis (spores). Spores are dispersed in an anhydrous state and become activated upon contact with water and swell, forming spherical cells. Spore cells then divide asymmetrically, and this asymmetry, in turn, aligns the first body axis that defines the position of the first meristem from which the mature plant body develops (*4*–*6*). The cues that orient the spore cell asymmetry are unknown.

A dry *M. polymorpha* spore is ∼7 to 10 μm in diameter and expands to about 20 μm within the first 24 hours after imbibition. After ∼28 hours, the spore nucleus moves from the cell centroid to the cell cortex at the future basal pole. There, the nuclear membrane breaks down, the mitotic spindle forms, chromosomes are separated at mitosis, and a new cell wall forms near the basal pole during cytokinesis, producing a small cell near the basal pole and a larger cell, which includes the entire apical hemisphere and most of the basal hemisphere. The large apical cell divides to form a population of cells from which a meristem forms, while the smaller basal cell differentiates into a germ rhizoid, which anchors the developing plant to the substrate (*7*–*9*). The movement of the nucleus to the basal pole requires reorganization of the microtubule and actin microfilament cytoskeletons, which provide both the direction and force for nuclear movement (*10*–*13*). However, neither the cue nor the molecular mechanism that orients spore cell asymmetry in *M. polymorpha* is known. Here, we report the discovery that a blue light cue perceived by the phototropin photoreceptor and its interaction partner NCH1 (NRL PROTEIN FOR CHLOROPLAST MOVEMENT 1) mediate the perception of this environmental signal.

## RESULTS

### Light positively regulates the division of the *M. polymorpha* spore

Dry *M. polymorpha* spores (Fig. 1A) became spherical with a diameter of ∼7 to 10 μm, within 12 hours of imbibition (Fig. 1D). By 24 hours after initiation of imbibition, spores were ∼20 μm in diameter, with dense cytoplasm and a nucleus located near the centroid (Fig. 1, B and D). Spores divided asymmetrically after ∼30 hours, forming a large apical germ cell and a smaller basal cell, separated by a new cell wall that was perpendicular to the apical-basal axis of the two-cell stage plant (Fig. 1, C to E) (*10*, *14*). The larger daughter cell was generative and proliferated into a mass of morphologically similar cells that formed the main body of a sporeling (Fig. 1C), on which a meristem developed de novo 8 to 10 days after plating in white light (*15*). The basal cell differentiated as a tip-growing rhizoid cell.

**Fig. 1.**
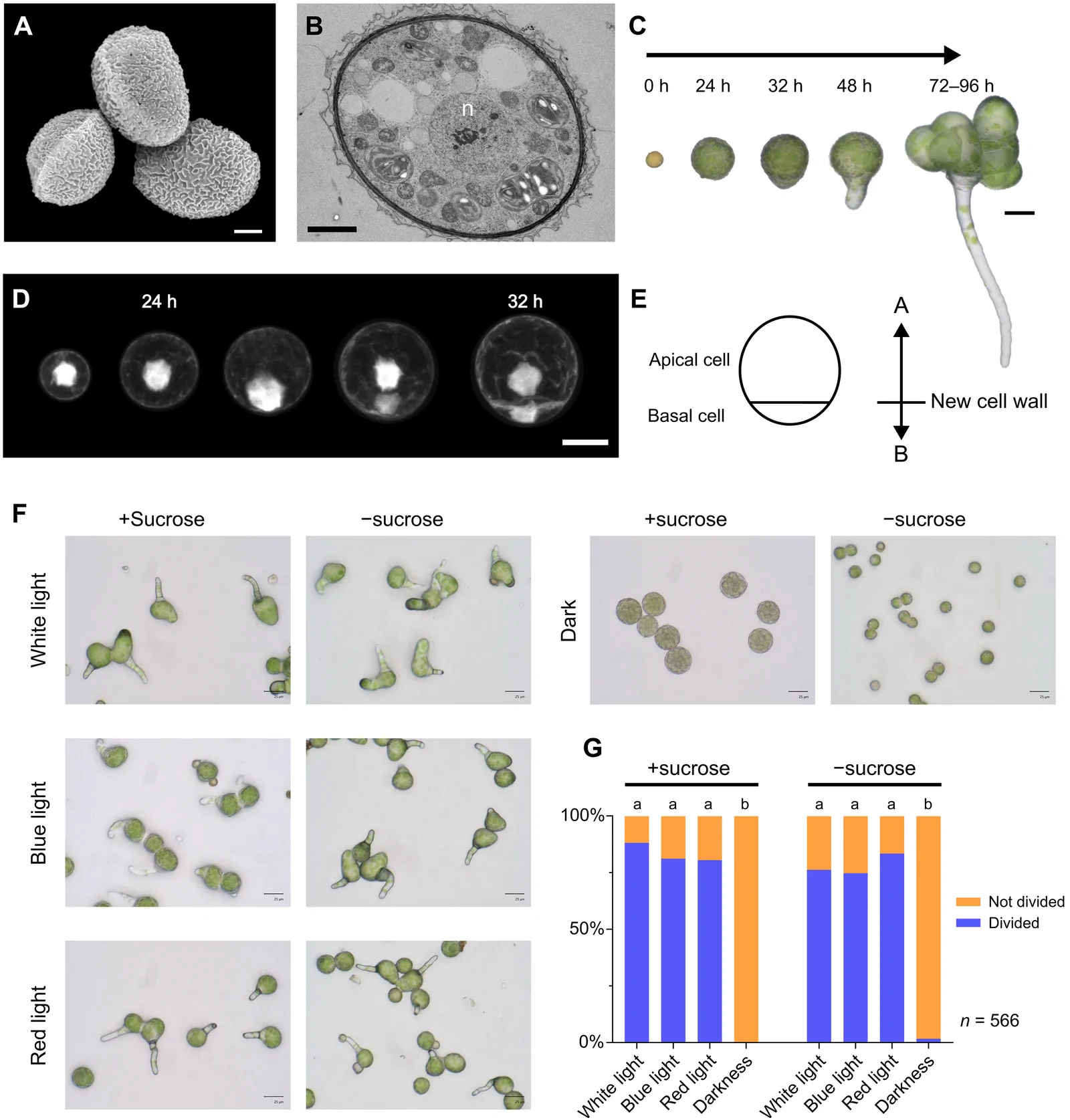
Light positively regulates the division of the *M. polymorpha* spore. (**A**) SEM image of dry *M. polymorpha* spores. Scale bar, 2 μm. (**B**) TEM image of a wild-type spore 24 hours (h) after initiation of imbibition, showing dense cytoplasm and a nucleus (n) located near the cell centroid. Scale bar, 3 μm. (**C**) Representative bright-field images of spores at the indicated times after plating. Scale bar, 20 μm. (**D**) Visualization of nucleus position in spores expressing nuclear and plasma membrane markers (*pro*Mp*ROP:mScarletI-N7* and *pro*Mp*UBE2:mScarletI-*At*LTI6b*). Spores were imaged after 12, 24, 29, and 32 hours. Maximum intensity projections (*xy*) of 3D z-stacks from five representative spores are shown. Scale bar, 10 μm. (**E**) Schematic of the two-cell spore stage following the first cell division, showing a larger apical cell and a smaller basal cell. The newly formed cell wall is oriented perpendicular to the apical-basal axis. (**F**) Spores grown for 2 days in darkness, white light (PFD = 50 to 60 μmol/m^2^ per second), blue light (PFD = 30 to 40 μmol/m^2^ per second, λ_max_ = 450 nm), or red light (PFD = 80 to 90 μmol/m^2^ per second, λ_max_ = 660 nm) on nutrient medium with or without sucrose. Scale bars, 25 μm. (**G**) Quantification of spore cell division after 48 hours. Proportions of dividing and nondividing spores (total *n* = 566) are shown for each condition. Chi-square tests of independence revealed significant effects of light treatment both with sucrose [χ^2^(3) = 161.7, *P* < 0.0001] and without sucrose [χ^2^(3) = 72.1, *P* < 0.0001]. Different letters indicate statistically significant differences (*P* < 0.05); identical letters indicate no significant difference.

To test the role of light in sporeling development, we grew spores in darkness, white light, red light, and blue light in nutrient medium without a sucrose supplement. Spore cells did not divide if grown in darkness for up to 7 days (2-day data, Fig. 1, F and G, and fig. S1A; 5-day data, fig. S1B; 7-day data, fig. S1C). By contrast, 76% of spore cells divided after 2 days in white light, 83% in red light, and 75% in blue light (Fig. 1, F and G, and fig. S1A). These data indicate that light is required for cell division (*16*). To test whether the lack of cell division in darkness was due to carbon starvation resulting from a lack of photosynthesis, we grew spores in nutrient medium supplemented with 1% (w/v) sucrose (Fig. 1F and fig. S1A). Dark-grown spores did not divide (0%) after 2 days, while spores grown in white (88%), red (80%), and blue (81%) light divided (Fig. 1G). These data indicate that light is required to stimulate spore division in the first days after imbibition and that monochromatic red or blue light alone is sufficient to initiate division. Dark-grown spores divided after 5 days (73%) and 7 days (86%) on sucrose-supplemented medium, indicating that spores can eventually divide in the absence of light (fig. S1, B and C) when supplemented with a carbon source, as previously reported (*16*). From these data, we conclude that the first cell division of the spore at ∼30 hours is dependent on light.

### Blue light orients the first cell division

We reasoned that either light, gravity, or both would control cell division orientation. To determine whether light directs nuclear migration that positions and orients cell division, we grew spores under unidirectional light on vertically oriented medium plates. The larger apical cell developed on the side nearest the incident light, while the smaller basal cell developed on the relatively shaded side (opposite the incident light direction) (Fig. 2A). The perpendicular orientation of the new cell wall relative to the incident light results in the apical cell being located on the illuminated side and the basal cell on the relatively shaded side. We measured the angles between the direction of incident light and the apical-basal axis (fig. S2A). The angles were close to 0°, indicating that the light vector aligns with the apical-basal axis in spores grown under unidirectional white light (Fig. 2A). These data suggest that light orients the asymmetric cell division in the spore cell. However, in these experiments, the light and gravity vectors were aligned, making it impossible to distinguish between the two.

**Fig. 2.**
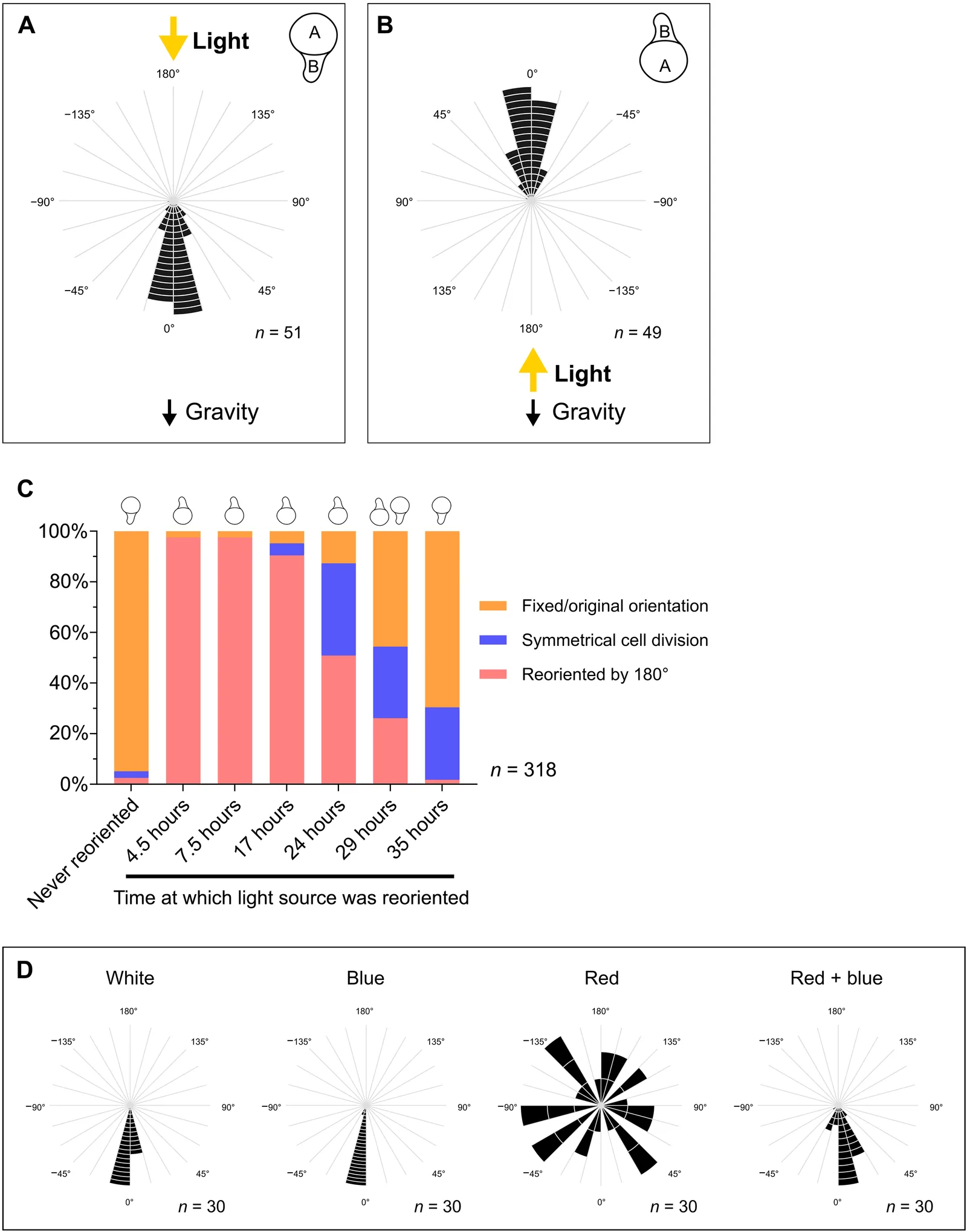
Blue light orients the first cell division. (**A**) Radial histogram of wild-type spores (*n* = 51) grown for 48 hours in continuous unidirectional white light (PFD = 50 to 60 μmol/m^2^ per second) on vertically oriented medium plates. Illumination from above aligned light and gravity vectors. The new cell wall formed perpendicular to the incident light, with the apical cell on the illuminated side and the basal cell on the relatively shaded side. (**B**) Radial histogram of wild-type spores (*n* = 49) grown for 48 hours in continuous unidirectional white light (PFD = 50 to 60 μmol/m^2^ per second) on vertically oriented medium plates, with light and gravity vectors opposed (illumination from below). The new cell wall formed perpendicular to the incident light and the basal cell developed on the relatively shaded side. These data indicate that light, not gravity, orients asymmetric division. (**C**) Reorientation experiments show that division orientation remains labile until shortly before division. Spores (*n* = 318) were grown in continuous unidirectional white light (PFD = 50 to 60 μmol/m^2^ per second), and the direction of light was reversed by 180° at different time points; all samples were analyzed after 48 hours. In controls (never reoriented), the basal cell formed on the relatively shaded side. (**D**) Spore orientation assays under different light conditions show that blue light orients the first cell division. Spores were grown for 48 hours in continuous white (PFD = 50 to 60 μmol/m^2^ per second), red (PFD = 80 to 90 μmol/m^2^ per second, λ_max_ = 660 nm), blue (PFD = 30 to 40 μmol/m^2^ per second, λ_max_ = 450 nm), or red + blue light in 3D agar culture in chambered microscopy slides. All orientation experiments were performed using wild-type spores expressing the fluorescent marker *pro*Mp*SYP13A:mCitrine:*Mp*SYP13A* to visualize cell division.

To test whether gravity contributes to the orientation of spore cell asymmetry (*17*–*19*), we aligned the light and gravity vectors at 180° to each other by shining light from below. If gravity defined the direction of asymmetry, then we would expect the smaller basal cell to develop on the lower, illuminated side and the larger apical cell to develop on the upper, shaded side. On the other hand, if light oriented asymmetry, then we would expect the smaller basal cell to develop on the upper shaded side and the large apical cell to develop on the lower illuminated side. When illuminated from below, the smaller basal cell formed on the upper shaded side, and the new cell wall was oriented perpendicular to the incident light (Fig. 2B). These data do not support the hypothesis that gravity directs asymmetry. Instead, they indicate that light directs the development of cellular asymmetry that orients asymmetric division of the spore. Although these data do not provide evidence for a role of gravity in orienting nuclear migration and cellular asymmetry, it remains possible that a relatively weak effect of gravity is masked by the stronger effect of light under these experimental conditions.

To understand when the light signal acts during the orientation of spore asymmetry, we tested the stability of light-induced asymmetry. We grew spores for varying durations in white incident light from one direction before changing the direction of the incident light by 180°. Measurements were taken after a total of 48 hours of light exposure for all treatments. In controls where light was applied from one direction for the duration of the experiment, cell division was asymmetric, and the smaller basal cell formed on the relatively shaded side. When the direction of light was reversed by 180° at different time points, up to 29 hours, the position of the smaller basal cell formed by asymmetric cell division was also reversed (Fig. 2C and fig. S2B). This indicates that the light-directed asymmetry is labile and changes if the direction of incident light changes until ∼29 hours. Reversal of the light direction at 24 and 29 hours resulted in a mixed population where the smaller basal cells developed at either pole, or, in some cases, division was symmetrical, forming two equal-sized cells (Fig. 2C and fig. S2B). Spore germination and cell division are asynchronous, and nuclear migration occurs between 24 and 29 hours (*10*). The mixed orientations observed at these late time points are consistent with developmental asynchrony within the spore population, such that individual spores were at different stages when the light direction was altered. Reversal of the light direction at 35 hours did not affect the position of the smaller basal cell—the smaller cell always developed on the shaded side relative to the direction of first illumination (Fig. 2C and fig. S2B). Since asymmetric cell division occurs at around 30 hours, we conclude that the cell asymmetry remains labile until shortly before cell division.

To determine which light wavelengths direct cell wall orientation during the development of cellular asymmetry, we grew spores in white, blue, and red light and determined the position of the smaller basal cell and the new cell wall orientation. For the spore orientation assays, we built a custom illumination system (fig. S2C and data S1, “Lightbox”) in which spores cultured in 0.4% agar in chambered microscopy slides were illuminated by directional light, such that the direction of incident light was perpendicular to the gravity vector. This system allowed us to image and measure cell division orientation of spores in three-dimensional (3D) space. In white light, the smaller basal cell developed on the relatively shaded side, and the new cell wall was oriented perpendicular to the incident light (Fig. 2D). In blue light, the smaller basal cell also developed on the relatively shaded side, and the new cell wall was oriented perpendicular to the incident light (Fig. 2D). However, in red light, the smaller basal cell was randomly positioned, and the new cell wall was randomly oriented relative to the incident light (Fig. 2D). When we grew spores in red light supplemented with blue light, they oriented similarly to those grown in monochromatic blue light, with the smaller basal cell forming on the shaded side and the new cell wall oriented perpendicular to the incident light, further underpinning the role of blue light in orienting spore cells (Fig. 2D). We measured the angles between the direction of incident light and the apical-basal axis (fig. S2A) under the four tested light conditions: white, blue, red, and combined red and blue light. Significant directional clustering occurred in both white and blue conditions (Rayleigh test, *P* < 0.001, mean resultant length *r* = 0.99 and 0.98, respectively), as did the combined red and blue light treatment (*P* < 0.001, *r* = 0.94). In contrast, angles of the spores grown in red light were not clustered (*P* = 0.998, *r* = 0.009). Watson-Wheeler tests revealed significant differences in angular distributions among all groups (multigroup test, *P* < 0.001), with all pairwise comparisons also showing statistically significant differences (*P* < 0.005).

To further test the role of light in orienting cell division, we determined the orientation of cell division in those spores that divided after 7 days in the dark on sucrose-supplemented medium (fig. S1, B and C). The position of the smaller basal cell was not aligned and was random as observed in spores grown for 48 hours in red light (fig. S2D). The absence of a consistent orientation in both red light and darkness further supports the conclusion that gravity does not provide the primary orienting cue for cellular asymmetry. These data indicate that blue light provides the directional cue that orients the asymmetry of cell division. While red light stimulates cell division, it does not orient cell asymmetry.

### Mpphot-mediated blue light sensing orients the first cell division

Because blue light was required to orient the asymmetric cell division in the spore (Fig. 2), we hypothesized that a blue light receptor mediated this response (*20*–*22*). To identify the blue light photoreceptor responsible for orienting spore cell division, we generated transcriptomes from sporelings at 0, 15, and 30 hours after imbibition and performed RNA sequencing. mRNA levels of several blue light photoreceptors—*PHOTOTROPIN* (Mp*PHOT*), *CRYPTOCHROME* (Mp*CRY*), and *FLAVIN-BINDING KELCH REPEAT F-BOX PROTEIN* (Mp*FKF*)—were abundant (Fig. 3A). We hypothesized that Mpphot would regulate the direction of nuclear movement (*23*) because of its described role in directing chloroplast movement (*24*–*27*). We follow the phototropin nomenclature proposed by Briggs *et al.* (*28*).

**Fig. 3.**
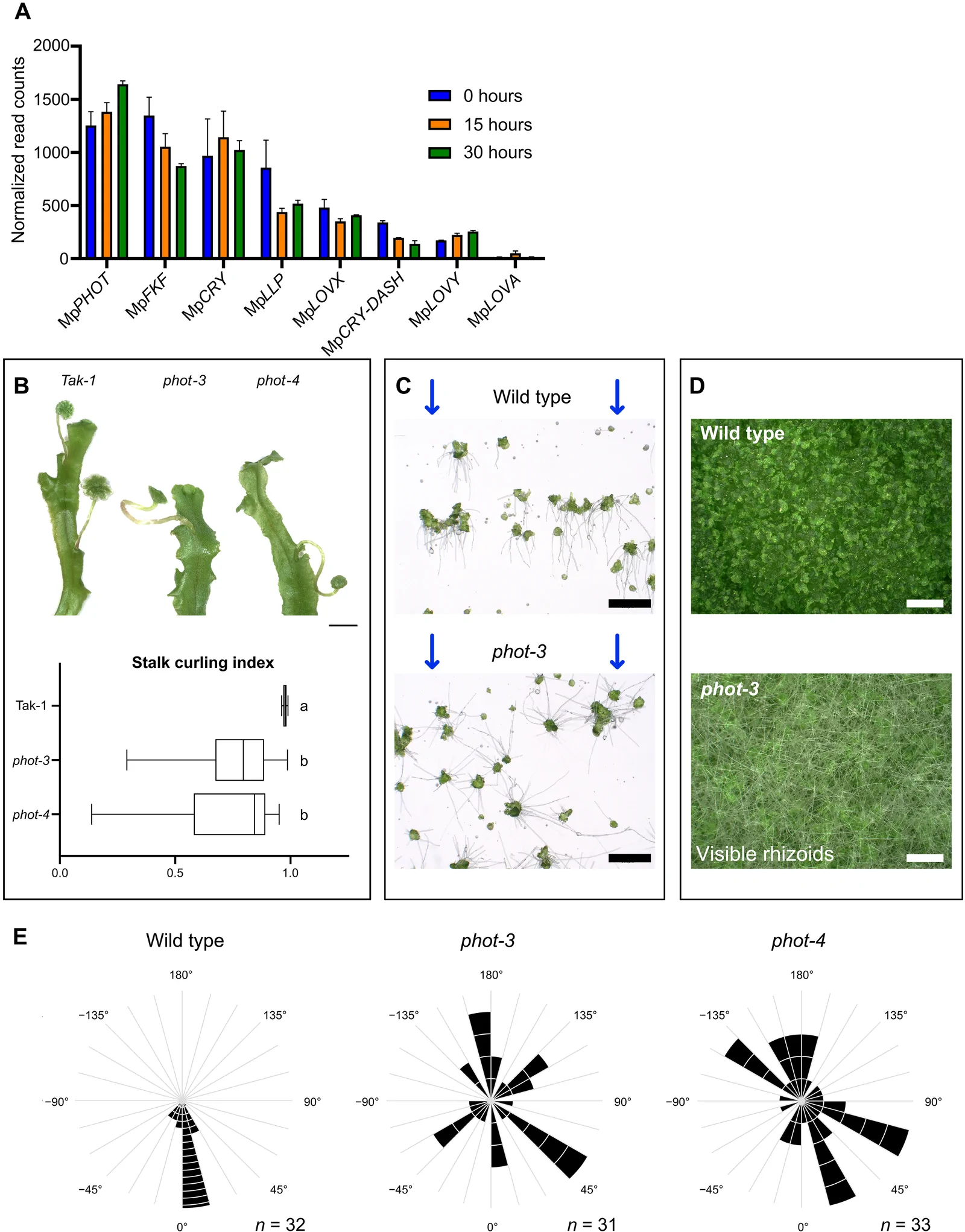
Mpphot-mediated blue light sensing orients the first cell division. (**A**) Blue light receptor genes are expressed early during spore development. Shown are normalized mRNA read counts at 0 hours (blue), 15 hours (orange), and 30 hours (green); two biological replicates per time point; mean with SD. (**B**) Tropic growth of gametangiophore stalks is defective in Mp*phot* mutants. Stalk curling was quantified in 8-week-old plants grown under white light (50 μmol/m^2^ per second) supplemented with far-red light (45 μmol/m^2^ per second) in a long-day regime (16-hour light/8-hour dark) at 23°C; *n* = 46 across three genotypes. Scale bar, 5 mm. Data were tested for normality (Shapiro-Wilk); as assumptions were not met, a Kruskal-Wallis test with Dunn’s multiple comparisons was performed. Different letters indicate significant differences (*P* < 0.05); identical letters indicate no significant difference. (**C**) Rhizoids of Mp*phot* mutant sporelings are defective in negative phototropism in blue light (PFD = 30 to 40 μmol/m^2^ per second, λ_max_ = 450 nm). In wild type, rhizoids of 10-day-old sporelings grew away from the blue light source, whereas mutant rhizoids grew in many directions. Scale bars, 1 mm. (**D**) Rhizoids of 11-day-old Mp*phot* mutant sporelings grew in many directions in white light. Under standard white light conditions (PFD = 50 to 60 μmol/m^2^ per second) on horizontal plates, rhizoids were visible in mutants but not in wild type. Spores were densely plated and imaged from above. Scale bars, 2 mm. (**E**) Mpphot is required to orient asymmetric cell division. Wild-type and Mp*phot* mutant spores expressing *pro*Mp*SYP13A*:*mCitrine*:Mp*SYP13A* were grown for 48 hours in continuous blue light (PFD = 30 to 40 μmol/m^2^ per second, λ_max_ = 450 nm).

To test whether Mp*phot* mutants are defective in tropic growth, we characterized tropic responses in gametangiophore stalks in independent mutant lines (*29*)*.* Tropic growth was defective in Mp*phot* mutants: Wild-type gametangiophore stalks grew straight and erect toward the light source. By contrast, Mp*phot* mutant gametangiophore stalks grew in different directions, resulting in curled stalk morphology (Fig. 3B). Stalk curling was quantified using a stalk curling index (SCI) defined as the ratio between the shortest and within-tissue distances from the stalk base to the base of the antheridial head. The index of straight stalks approaches 1 but is lower in curved stalks. The average index of wild type was ∼0.98, whereas the index of Mp*phot* mutants was ∼0.74 (Fig. 3B). The defective tropism of gametangiophore stalks in Mp*phot* mutants is consistent with the hypothesis that tropic responses are defective in these mutants.

Negative phototropism was also defective in Mp*phot* mutants. In wild type, rhizoids of 10-day-old sporelings grew away from the blue light source, while Mp*phot* mutant rhizoids were insensitive to light direction and grew in all directions (Fig. 3C). Similarly, under standard white light conditions on horizontal plates, rhizoids were visible in 11-day-old Mp*phot* sporelings but not in wild type when densely plated spores were imaged from above (Fig. 3D). The defective gametangiophore growth and defective rhizoid negative phototropism in Mp*phot* mutants demonstrate that Mpphot is required for both tropic growth and negative phototropic responses and that blue light signaling is defective in the Mp*phot* loss-of-function mutants.

To determine whether Mpphot is required to orient cell division asymmetry relative to the direction of incident blue light, we compared the position of the smaller basal cell and the orientation of the new cell wall that forms in unilateral light in wild type and Mp*phot* mutants. The smaller basal cell developed on the shaded side of the two-cell sporeling, and the new cell wall was oriented perpendicular to the direction of incident light in the wild type (Fig. 3E). By contrast, in Mp*phot* mutants, the smaller basal cell developed in random positions, and the orientation of the new cell wall was random with respect to the direction of light (Fig. 3F). We measured the angles between the direction of incident light and the apical-basal axis. Wild type clustered at consistently low angles, indicating that they all were oriented in the same direction (Rayleigh test, *P* < 0.001, mean resultant length *r* = 0.95), while Mp*phot-3* and Mp*phot-4* mutants were randomly oriented (*P* > 0.5, *r* = 0.12 and 0.14, respectively). Watson-Wheeler tests revealed significant differences in angular distributions between wild type and both mutants (*P* < 0.001) but not between Mp*phot-3* and Mp*phot-4* (*P* = 0.99). These data indicate that the position of the smaller basal cell and the orientation of the new cell wall were defective in Mp*phot* mutants. We conclude that Mpphot is required for the development of blue light–directed cell asymmetry that orients asymmetric cell division of the spore.

### MpNCH1 mediates Mpphot-dependent orientation of asymmetric cell division

To identify proteins involved in Mpphot-mediated blue light signaling, we performed in vivo proximity labeling using a Mpphot–miniTurbo(ID)–yellow fluorescent protein (YFP) fusion protein. Biotinylated proteins from two independent *pro*Mp*PHOT*:Mp*PHOT*-*miniTurbo-YFP* lines were compared with those from plasma membrane–localized control (*30*) (*pro*Mp*PHOT*:Mp*PIP2*-*miniTurbo-YFP*), as Mpphot is associated with the plasma membrane (*24*), and the wild-type control [Takaragaike-1 (Tak-1)]. All constructs were introduced into the same genetic background, Tak-1, by thallus transformation. Proteins biotinylated in the vicinity of Mpphot were identified by mass spectrometry. This identified 547 to 550 proteins that were significantly enriched (log_2_ fold change > 2, *P* <0.05) for two independently tested Mpphot-miniTurbo-YFP lines against Tak-1, respectively (Fig. 4A and table S1). From the enriched candidates, we prioritized 11 proteins for further analysis based on their expression in the one-cell spore stage (≤30-hour transcriptomes) (Fig. 3A) and their predicted roles in signal transduction (candidates highlighted in Fig. 4A). To test whether any of the 11 proteins were required for blue light–oriented division of the spore cell, we generated a total of 137 mutant lines carrying loss-of-function mutations in the respective genes (fig. S4A).

**Fig. 4.**
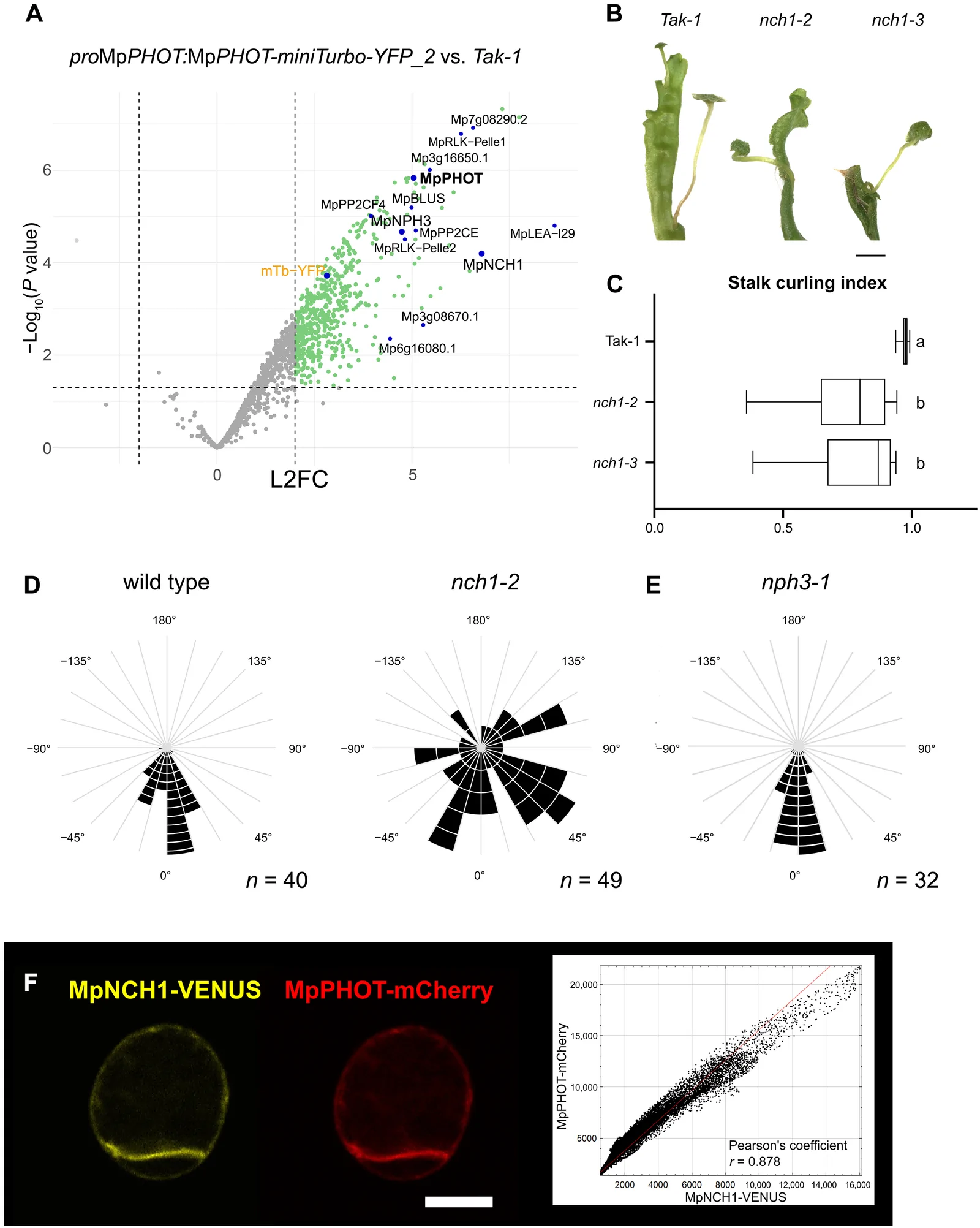
MpNCH1 mediates Mpphot-dependent orientation of asymmetric cell division. (**A**) Proximity labeling–based proteomics identifies proteins in close proximity to Mpphot. Mpphot-miniTurbo(ID)-YFP fusion proteins were used to biotinylate proteins in continuous white light, followed by mass spectrometry. The volcano plot shows differential enrichment relative to wild type (Tak-1) from triplicate samples [*P* < 0.05, log_2_ fold change (L2FC) > 2]. Additional comparisons of independent experimental lines with plasma membrane and wild-type controls are provided in table S1. (**B**) Tropic growth of gametangiophore stalks is defective in Mp*nch1* mutants. Scale bar, 5 mm. (**C**) Quantification of stalk curling in 8-week-old plants grown under white light (50 μmol/m^2^ per second) supplemented with far-red light (45 μmol/m^2^ per second) in a long-day regime (16-hour light/8-hour dark) at 23°C; *n* = 37 across three genotypes. Data were tested for normality (Shapiro-Wilk); non-normal data were analyzed using a Kruskal-Wallis test with Dunn’s multiple comparisons. Different letters indicate significant differences (*P* < 0.05); identical letters indicate no significant difference. (**D**) MpNCH1 is required to orient asymmetric cell division. Wild-type and Mp*nch1* spores expressing *pro*Mp*SYP13A*:*mCitrine*:Mp*SYP13A* were grown for 48 hours in continuous blue light (PFD = 30 to 40 μmol/m^2^ per second, λ_max_ = 450 nm). (**E**) MpNPH3 is not required for orientation of asymmetric cell division. Mp*nph3* spores grown under the same conditions showed wild type–like orientation. (**F**) MpNCH1-VENUS and Mpphot-mCherry colocalize in spores. The cytofluorogram shows pixel intensity correlation from a single plane of an individual cell (thresholded; Gaussian blur, radius = 2). Signals are strongly correlated (Pearson’s *r* = 0.878), indicating overlapping localization. Spores were grown for 36 hours on cellophane-covered medium under standard white light conditions (PFD = 50 to 60 μmol/m^2^ per second), fixed, cleared, and imaged by confocal microscopy. Scale bar, 10 μm.

Next, we characterized light responses in the thallus and gametangiophores. While some mutants developed defective thalli, only mutants in one gene—Mp*NRL PROTEIN FOR CHLOROPLAST MOVEMENT 1* (Mp*NCH1*)—developed the curled gametangiophore phenotype characteristic of defective blue light signaling, as observed in the Mp*phot* mutants (Fig. 4B). The stalk curling index (SCI) of Mp*nch1* mutants was ∼0.77 (Fig. 4C) and similar to Mp*phot* mutants (Fig. 3C), whereas the wild type developed an index close to 1 (∼0.97; Fig. 4C). These data indicate that blue light signaling is defective in Mp*nch1* mutants.

We then compared the position of the smaller basal cell and new cell wall orientation in Mp*nch1* mutants and wild type. In wild type, the new basal cell formed on the shaded side, and the new cell wall was oriented perpendicular to the incident light (Fig. 4D). However, in Mp*nch1* mutants, the smaller basal cell developed in random positions relative to the direction of illumination, and the new cell wall was randomly oriented (Fig. 4D). We measured the angles between the direction of incident light and the apical-basal axis. The orientation angles of the wild type are clustered (Rayleigh test, *P* < 0.001, *r* > 0.9), whereas the orientation of the axis is largely random with only weak residual directionality (*r* = 0.34) in Mp*nch1* mutants. Consistent with this, the angular distributions of wild type and Mp*nch1* mutants differ significantly (Watson-Wheeler test, *P* < 0.001). These data indicate that Mp*NCH1* is required for blue light–directed asymmetry that orients asymmetric cell division in the spore.

MpNCH1 is a member of a family of NRL [NONPHOTOTROPIC HYPOCOTYL 3 (NPH3)/RPT2-like] proteins that have been implicated in Mpphot-mediated blue light signaling (*31*). MpNPH3 is also an NRL protein family member that functions in blue light signaling (*32*). To test whether MpNPH3 is required for blue light–oriented spore cell division, we generated lines carrying mutations in Mp*NPH3* and compared the positioning of the smaller basal cell and new cell wall orientation in Mp*nph3* loss-of-function mutants with wild type. Both the position of the small basal cell and the orientation of the new cell wall in the Mp*nph3* spores were wild type like (Fig. 4E). The orientation angles are clustered in Mp*nph3*, similar to wild type (Rayleigh test, *P* < 0.001, *r* > 0.9). These data indicate that Mp*NPH3* is not required for the orientation of cell asymmetry in the *M. polymorpha* spore.

If Mpphot and MpNCH1 interact during the establishment of cell asymmetry in the spore, we predicted that the proteins should colocalize during this process. Subcellular localization of Mpphot and MpNCH1 has been reported previously in independent studies in separate lines (*24*, *31*). To test the hypothesis on subcellular colocalization, we generated marker lines expressing *pro*Mp*NCH1:*Mp*NCH1-VENUS* and *pro*Mp*PHOT:*Mp*PHOT-mCherry* in the same spore. Spores were grown for 24 and 36 hours under standard white light, then fixed, and cleared to remove chloroplast autofluorescence. Mpphot-mCherry and MpNCH1-VENUS colocalized at the cell surface (Fig. 4F and fig. S4B). These data are consistent with Mpphot and MpNCH1 acting in the same pathway to regulate blue light–oriented cell asymmetry in the *M. polymorpha* spore.

## DISCUSSION

We found that Mpphot-mediated blue light signaling aligns the cellular asymmetry that orients the first division of the spore and sets up the first developmental axis of the *M. polymorpha* body plan. Mpphot-mediated blue light signaling that directs the orientation of cellular asymmetry requires MpNCH1 but not the related MpNPH3 NRL protein (*31*). In *Arabidopsis*, NRL proteins such as NPH3 and RPT2 act as key components of phototropin signaling during phototropism, where NPH3 is essential for hypocotyl bending and RPT2 contributes to both phototropism and chloroplast movement. NCH1 is primarily associated with chloroplast movement pathways and is not a central component of phototropism (*32*). Thus, whereas NCH1 does not play a major role in phototropism in *Arabidopsis*, our results indicate that in *Marchantia* MpNCH1 is required for Mpphot-mediated orientation of spore cell asymmetry, suggesting a role for MpNCH1 in light-directed development in this system.

Our data demonstrate that the orientation of cell asymmetry and subsequent body axis orientation depend on an external blue light cue from the environment. This externally directed cellular asymmetry positions the germ rhizoid on the shaded side of the spore. The rhizoid cell anchors the plant to the substrate during its early development (*4*). The apical cell is on the illuminated side, divides, and forms a photosynthetically active sporeling that develops a meristem from which the plant body develops (*15*).

In land plants, the diploid zygote develops within the maternal tissues in which the egg cell formed: within the archegonium in bryophytes, lycophytes, monilophytes, and gymnosperms and within the embryo sac in angiosperms (*1*). Signals from the parental tissues polarize the egg and zygote (*3*, *33*). By contrast, haploid spores that form in bryophytes and monilophytes develop in the environment and independent of the plant on which they are produced. The cues that orient development do not come from surrounding tissues (*11*). We showed that light positively regulates the division of *M. polymorpha* spores and that either monochromatic red or blue light alone is sufficient to initiate cell division. This is consistent with previous work in *M. polymorpha*, which reported that light—including green light—and photosynthetic activity promote spore germination and led to the conclusion that photosynthesis contributes to the induction of germination (*16*). In other free-sporing plants, including several fern species, blue light has been shown to inhibit spore germination, as reviewed by Suo *et al.* (*34*). Instead, we show here that blue light orients the symmetry-breaking event that occurs in spores in the first 30 hours of growth before cell division. This cell asymmetry orients the first cell division, the first body axis and, subsequently, the negative phototropic growth of the germ rhizoid. In this way, Mpphot-mediated blue light signaling orients the development of the *M. polymorpha* body, tethering the plant to its substrate.

The demonstration that both Mpphot and MpNCH1 are required to direct cell asymmetry in the spore cell suggests that a canonical Mpphot signaling pathway regulates this process. In addition to MpNCH1, proximity labeling revealed several proteins previously linked to phototropin signaling, including a BLUS-like protein (*35*) and multiple kinases and phosphatases (Fig. 4A and fig. S4A). These associations are consistent with engagement of a broader phosphorylation-dependent signaling framework. Nevertheless, our functional analyses identify MpNCH1 as a key downstream component required for orienting spore cell asymmetry. This discovery reveals how plants exploit a directional environmental cue to align activities in the single totipotent cell from which the multicellular haploid plant develops.

## MATERIALS AND METHODS

### Plant material and growth conditions

Wild-type accessions of *M. polymorpha*, Tak-1 (male) and Tak-2 (female) (*36*), together with the respective mutant lines, were cultivated on sterile solid medium. The growth medium consisted of half-strength Gamborg’s B5 (*37*) supplemented with MES hydrate (0.5 g/liter), 1% (w/v) sucrose, and 1% (w/v) plant agar, adjusted to pH 5.5 before autoclaving. The plants were cultivated at 23°C under continuous white light illumination in standard light-emitting diode (LED) growth chambers at the institute or in growth chambers manufactured by Poly Klima Climatic Grow Systems [Hofmann Kühlung Company; firmware version 03.10.10, controller: WAGO 750-8212 PFC200 G2 2ETH R]. For dark treatment controls, plates were wrapped twice with aluminum foil, transferred into a light-impermeable container, and maintained in the same growth chamber.

Unless otherwise stated in the respective figures, the following light regimes were applied: standard continuous white light at a photon flux density (PFD) of 50 to 60 μmol/m^2^ per second; blue light at ∼30 to 40 μmol/m^2^ per second (λ_max_ = 450 nm); and red light at ∼80 to 90 μmol/m^2^ per second (λ_max_ = 660 nm). Dark controls were prepared as described above and kept at 23°C in the growth chamber. Light intensities were quantified using a Spectral PAR Meter PG100N (UPRtek).

For gametangiophore induction, plants were transferred to soil in SacO_2_ Microbox containers containing an autoclaved 3:1 mixture of Neuhaus N3 compost and vermiculite and grown under white light (50 μmol/m^2^ per second) supplemented with far-red light (45 μmol/m^2^ per second) in a long-day regime (16-hour light/8-hour dark) at 23°C. Plants were illuminated from above, as the LED light source was mounted at the top of the growth chamber.

### Scanning electron microscopy of dry *M. polymorpha* spores

Sporangia from a cross between Tak-1 and Tak-2 were collected and dried over silica gel at room temperature for 3 to 4 weeks. Dry *M. polymorpha* spores were mounted on scanning electron microscopy (SEM) stubs and sputter coated with gold for 30 s using a JEOL JFC-2300HR sputter coater to generate a conductive surface layer.

Samples were imaged using a JEOL IT300 scanning electron microscope equipped with a LaB6 filament. Secondary electron and backscattered electron detectors were used for image acquisition. SEM imaging was performed at the Core Facility Cell Imaging and Ultrastructure Research (CIUS), University of Vienna, member of the Vienna Life-Science Instruments (VLSI).

### Transmission electron microscopy of *M. polymorpha* spores

*M. polymorpha* spores were cultured on cellophane-covered medium plates under continuous white light conditions for 24 hours after plating. The spores were then washed off the cellophane and immediately prefixed in 0.5% (w/v) paraformaldehyde and 0.05% (v/v) glutaraldehyde in 0.1 M PHEM buffer for 10 min. Samples were washed twice in PHEM buffer, transferred to Gamborg medium supplemented with 10% (w/v) bovine serum albumin (fraction V), and cryo-immobilized using a Wohlwend HPF Compact 01 high-pressure freezer.

Freeze substitution was performed in a Leica EM AFS automated system over 3 days at −90°C in acetone containing 0.1% (w/v) tannic acid and 0.5% (v/v) glutaraldehyde. After substitution, samples were warmed to −20°C over 12 hours, washed in precooled acetone, and incubated in 2% (w/v) osmium tetroxide in acetone for 24 hours for infiltration and postfixation. Samples were then warmed at ∼5°C/hour to 4°C, rinsed, incubated for 2 hours at 4°C, brought to room temperature, and washed further in acetone.

Samples were infiltrated with Agar 100 epoxy resin through a graded resin:acetone series and polymerized in pure resin at 60°C for 48 hours. Ultrathin sections (70 nm in nominal thickness) were cut using a Leica UCT ultramicrotome and poststained with 2% (w/v) uranyl acetate and Reynolds’ lead citrate.

Transmission electron microscopy (TEM) imaging was performed on an FEI Morgagni 268D transmission electron microscope equipped with a tungsten filament emitter and a MegaView III charge-coupled device camera (Olympus-SIS). The instrument was operated at 80 kV (nominal maximum of 100 kV). Images were acquired using Morgagni software (v3.0) and iTEM (v5.0). Digital images were recorded at ×7100 magnification with an image size of 1376 pixels by 1032 pixels (16-bit), corresponding to a pixel size of 9.151 nm.

TEM imaging of *Marchantia* spores was carried out at the Electron Microscopy Facility, Vienna BioCenter Core Facilities (VBCF), member of the Vienna BioCenter (VBC), Vienna, Austria.

### Bright-field imaging of plants and spores

Plants and spores were imaged using a Keyence VHX-7000 digital bright-field microscope equipped with VH-Z00R/Z00T and VH-ZST lenses and a VHX-7020 camera. Where necessary, nonspecific background was removed, and images were cropped to appropriate dimensions for figure preparation.

### Spore generation, culture, and imaging

Crosses between male and female plants were performed by collecting sperm from antheridiophores in sterile water for 15 min and transferring the suspension onto archegoniophores. Fully developed sporangia were collected 4 to 5 weeks after fertilization, placed in sealed polypropylene microboxes (OV80 + OVD80) with micropore tape, and dried over silica gel (*36*, *38*). After a 4-week desiccation period, sporangia were stored at −70°C until use.

For germination, frozen sporangia were thawed and gently crushed in 0.1% (w/v) sodium dichloroisocyanurate (NaDCC; Sigma-Aldrich, catalog no. 218928) for 1 to 2 min. Spores were pelleted (13,000*g* for 3 min), washed twice with sterile water, and resuspended in sterile water.

Spores were plated on sterile medium consisting of half-strength Gamborg B5 supplemented with MES hydrate (0.5 g/liter) (pH 5.5), containing 1% sucrose or no sucrose, and solidified with 1% plant agar or 1% phytoagar. Cultures were grown at 23°C under continuous illumination. For dark controls, plates were wrapped twice in aluminum foil, placed in a light-tight box, and maintained in the same growth chamber.

For spore orientation assays, wild-type and mutant spores expressing *pro*Mp*SYP13A:mCitrine:*Mp*SYP13A* were embedded in half-strength B5 medium [B5 (1.5 g/liter), MES (0.5 g/liter), and 1% sucrose (pH 5.5)] solidified in 0.4% plant agar. The agar medium was cooled to near-solidification before mixing with spores to generate a homogeneous 3D culture, which was solidified in chambered microscopy slides (Ibidi, catalog no. 80286). Slides were placed in a custom-built illumination system (Lightbox).

All procedures involving spores for light assays were performed under dim green light in a sterile laminar flow hood.

### Custom-built illumination system (Lightbox) for spore orientation experiments

#### Spore orientation assays

Spore orientation assays were performed using the custom-built Lightbox system described in this section. This setup enables controlled directional illumination of spores in 0.4% 3D agar culture in chambered microscopy slides. In these experiments, the direction of incident light was perpendicular to the gravity vector.

#### Software and control

The Lightbox control software was developed mainly by Sebastian Seitner (PlantS Facility, GMI Vienna). The system comprises four independent chambers, each equipped with a dedicated sample holder and LED module (fig. S2C and data S1). All modules can be controlled independently.

Illumination regimes are defined as schedules and uploaded to the device via USB-C in JSON (JavaScript Object Notation) format. Each schedule specifies the sequence, duration, and intensity of light conditions for each chamber. The uploaded schedules are selected via an integrated user interface consisting of a rotary control and LCD display. Each module contains 16 high-power LEDs (OSRAM), covering four spectral ranges: white (3500 K), blue (455 nm), red (657 nm), and far-red (727 nm).

Light intensity is regulated using pulse width modulation (PWM) at ∼30 kHz. PWM enables stable low-intensity output without shifting emission spectra. LEDs of each color are arranged in two electrically independent groups. These groups are driven with a 180° phase offset. At intensities above 50%, at least one group remains active at any time. This reduces temporal fluctuations in light delivery.

#### Hardware

The Lightbox hardware was developed by Martin Colombini and the Mechanical Engineering Center (VBC). The system consists of a base plate, a control unit, and a light-tight enclosure containing four independent chambers. Each chamber fits one two-well chambered coverslip from Ibidi (catalog no. 80286).

The enclosure includes a rear panel supporting the LED boards, a front panel with an access door, structural side panels, internal partitions, and top covers. Samples are inserted through the front door and positioned precisely within each chamber. Illumination parameters are set via the external control unit.

Thermal control is facilitated through active airflow: A top-mounted fan draws air from the base and exhausts it upward, cooling the LED assemblies. The rear LED compartment is covered by a removable panel to maintain airflow while allowing access for maintenance. Each chamber is additionally ventilated by side-mounted fans. Air enters through a filtered inlet, passes through a light-blocking labyrinth, and exits through a corresponding outlet. These structures prevent external light contamination.

Optical isolation between chambers is ensured by internal partitions, diaphragm elements, and sealing interfaces between the front panel and door. These features prevent light leakage and cross-illumination.

The structural components (base, front, rear, and side panels) are constructed from rigid polyvinyl chloride. Covers, partitions, and the door are made from anodized aluminum. Internal rails are aluminum with a sandblasted finish to reduce reflections. Interior panels consist of black acrylic. Components are assembled primarily using stainless steel screws.

#### Previous setup for spore orientation experiments

Initial (re)orientation and gravity experiments were performed using a simplified setup. Unidirectional illumination was provided by single LEDs mounted on a breadboard. To expose spores to unidirectional light, chambered microscopy slides (Ibidi, catalog no. 80286) containing spores were placed inside a 3D-printed cover box, in which all sides except one were enclosed by a light-proof black cover. This configuration allowed light to enter from only one side of the chamber, creating unidirectional illumination. The direction of incident light was perpendicular to the gravity vector.

The entire setup was placed in an opaque enclosure (a black cardboard box) for the duration of the experiment. Light intensity was measured using a Spectral PAR Meter PG100N (UPRtek).

### Spore orientation quantification—Angle calculation

Angles between the light vector and the apical-basal axis were measured using Fiji and visualized in RStudio. 2D or z-stacked confocal images were acquired from wild-type and mutant spores expressing the fluorescent marker *pro*Mp*SYP13A:mCitrine:*Mp*SYP13A* to visualize cell division. Images were oriented, such that the direction of incident light corresponded to the negative *y* axis.

In Fiji, the transverse cell wall of asymmetrically dividing sporelings was identified manually. Using the multipoint tool, the two junctions where the transverse wall meets the spore outline were marked sequentially, and *XY* coordinates were exported for each image. Angles were calculated in RStudio using the exported coordinate files. The apical-basal axis was defined as the vector orthogonal to the transverse cell wall. The angle between this axis and the light vector (negative *y* axis) was then computed.

All calculated angles from individual measurements for each genotype and experiment were used to generate binned radial histograms. Visualization was performed using the R packages circular, plotrix, and ggplot2.

### Fixation, clearing, and cell wall staining of spores

Spores expressing nuclear and plasma membrane markers (*10*) (*pro*Mp*ROP:mScarletI-N7* and *pro*Mp*UBE2:mScarletI-*At*LTI6b*; Fig. 2D) and spores expressing fluorescent reporters for colocalization (*pro*Mp*NCH1:*Mp*NCH1-VENUS* and *pro*Mp*PHOT:*Mp*PHOT-mCherry*; Fig. 4F) were cultivated on cellophane-covered standard medium with sucrose for the indicated time periods. Cellophane with spores was removed and submerged in 10% formalin containing 0.1% Brij L23 for 30 min. Fixed sporelings were pelleted (7000*g* for 3 min), washed in 1× phosphate-buffered saline, and cleared in ClearSee α solution [10% (w/v) xylitol, 15% (w/v) sodium deoxycholate, 25% (w/v) urea, supplemented with sodium sulfite anhydrous (6.3 mg/ml)] for ∼1 week in the dark with gentle agitation.

Cleared sporelings were mounted in ClearSee α and imaged by confocal microscopy (Zeiss LSM 880) using a 40×/1.2 LD LCI Plan-Apochromat water/glycerol differential interference contrast objective with silicone immersion oil matched to the refractive index of ClearSee α. For colocalization analysis (Fig. 4F), single-channel z-stacks (VENUS and mCherry) were processed in ImageJ/Fiji (*39*), and colocalization was quantified using JACoP (*40*).

For cell division analysis under different light conditions (Fig. 1G and fig. S1, A to C), the same fixation and clearing protocol was used with the addition of 0.2% Renaissance SR2200 applied overnight before imaging. The dye for cell wall staining was excited at 405 nm, and emission was collected between 420 and 500 nm using an upright wide-field microscope. Images were acquired with a scientific complementary metal-oxide semiconductor camera, and cell divisions were manually scored in Fiji (*39*).

### RNA sequencing and differential expression analysis of early spore development

To assess gene expression during early spore development, spores were harvested in two biological replicates at three timepoints: 0, 15, and 30 hours after plating on standard medium plates covered with cellophane. For each sample, six wild-type sporangia (derived from a cross of Tak-1 and Tak-2) were collected, and surface-sterilized with 1% NaDCC, followed by washing three times with sterile water. The spore suspension was plated and maintained under continuous white light (PFD of 50 to 60 μmol/m^2^ per second) for the indicated durations.

For collection, the cellophane with adhered spores was peeled from the medium, flash frozen in liquid nitrogen, and ground to a fine powder using a mixer mill (RETSCH MM 400) at 30 Hz for 4 min. Total RNA was extracted using the RNeasy Plant Mini Kit (QIAGEN) according to the manufacturer’s instructions, followed by Turbo deoxyribonuclease treatment to remove residual genomic DNA.

RNA sequencing was performed at the Wellcome Trust Centre for Human Genetics, University of Oxford, using an Illumina HiSeq 4000 platform generating 75–base pair paired-end reads. Each sample was sequenced in five technical replicates, with replicates distributed across five independent sequencing lanes. Raw reads were returned as fastq.gz files. Reads passing quality control (Phred +33 quality score of >30 assessed with FastQC) were trimmed by removing the first and last 10 bases, and adapters were removed using Trimmomatic (v0.32) (*41*). Paired-end reads were interleaved. Residual bacterial ribosomal RNA sequences were filtered with SortMeRNA (v2.1b) (*42*). Reads were error corrected using BayesHammer within SPAdes (v3.10.1) (*43*) and aligned to the *M. polymorpha* reference transcriptome (v3.1) (*44*) using Salmon (*45*).

Differential gene expression analysis was performed with DESeq2 (*46*). Transcript-level quantifications were summarized to gene-level counts using the tximport algorithm, and technical replicates were collapsed using the collapseReplicates function in DESeq2 package (*46*) in R. Gene annotations were obtained from the *M. polymorpha* reference genome (v3.1) (functional annotation downloaded from https://marchantia.info/download/v31/). Pairwise comparisons were conducted, with an adjusted *P* value (*P*_adj_) of <0.05 considered significant. Genes with a log_2_ fold change of <0 were classified as down-regulated, those with a log_2_ fold change of >0 were classified as up-regulated, and NA indicated no significant change.

Between 0 and 15 hours, 2953 genes were up-regulated, 2947 down-regulated, and 13,317 unchanged. Of the 2953 up-regulated genes, 360 were further up-regulated, 108 down-regulated, and 2485 unchanged at 30 hours. Of the 2947 down-regulated genes, 128 were up-regulated, 97 further down-regulated, and 2722 unchanged at 30 hours. Among the 13,317 unchanged genes between 0 and 15 hours, 290 were up-regulated, 85 down-regulated, and 12,942 remained unchanged at 30 hours. The raw sequencing dataset, as well as Salmon alignment output (DESeq2 input) were uploaded on the National Center for Biotechnology Information (NCBI) GEO repository with the accession number GSE336751. A table of normalized gene counts across all samples is provided in table S2.

### Mutant generation using CRISPR-Cas9 mutagenesis

CRISPR-Cas9 mutagenesis was performed as described in detail in Roetzer *et al.* (*29*), including single guide RNA (sgRNA) design and target site selection, plasmid generation, bacterial transformation, generation of transgenic lines, *Agrobacterium*-mediated transformation of *M. polymorpha* sporelings, and mutant screening and genotyping. Briefly, CRISPR-Cas9–mediated mutagenesis was used to generate mutant lines for Mp*NCH1* (Mp5g07060) and Mp*NPH3* (Mp5g17270). sgRNAs were designed using CHOPCHOP (*47*) or manually for regions not listed among the top hits based on gene sequences from marchantia.info (MpTak v6.1r2) (*48*). Each sgRNA consisted of a 20-nt target sequence, followed by a canonical NGG protospacer adjacent motif (PAM), and candidate sgRNAs were checked for uniqueness against the *M. polymorpha* genome. Schematic representations of the Mp*NCH1* and Mp*NPH3* genes and their sgRNA target sites are depicted in data S2.

sgRNA duplexes were cloned into OP-076 vectors (L2_lacZgRNA-Cas9-CsA) from the OpenPlant kit (*49*), carrying dual antibiotic resistance markers. Recombinant plasmids were introduced into *Escherichia coli* DH5α cells, verified by Sanger sequencing, and purified for transformation. Mutant lines were generated using spores derived from a cross between Tak-1 and Tak-2 and *Agrobacterium*-mediated transformation with strain GV3101 (*36*). Transformants were selected on appropriate antibiotic-containing medium.

Genomic DNA from transformants was extracted and polymerase chain reaction (PCR)–amplified across gRNA target sites. The PCR products were sequenced using Sanger sequencing to identify mutations, and lines harboring mutations predicted to generate premature stop codons were selected. Mutant lines were propagated, and genotypes were repeatedly confirmed. Mutant alleles of the Mp*NCH1* and Mp*NPH3* genes generated and used in this study, along with the predicted effects of their mutations on protein length, are presented in data S2.

### Stalk curling index

Stalk curling was quantified by calculating an SCI. For this analysis, individual stalks bearing antheridial heads were imaged under standardized conditions. For each genotype, four independent plants were used.

To determine the SCI, two distances between the stalk base and the base of the antheridial head were measured. First, the “real length” was determined by tracing the within-tissue path along the curvature of the stalk from its base to the base of the antheridial head. Second, the “shortest distance” was measured as the linear distance between these same two reference points by drawing a straight line.

The SCI was calculated as the ratio of shortest distance to real length. An SCI value approaching 1 indicates a straight stalk, whereas lower values reflect increasing degrees of curvature. Length measurements were performed using the measurement software of the Keyence microscope VHX-7000.

### Sporeling orientation assay

Spores were surface sterilized as described above and resuspended in sterile water (one sporangium per 500 μl). For vertical blue light assays (Fig. 3C and fig. S3A), 25 μl of the suspension was evenly distributed onto square plates containing standard growth medium [½-strength B5 Gamborg with MES hydrate (0.5 g/liter), 1% (w/v) sucrose, and 1% (w/v) plant agar (pH 5.5)]. All handling before illumination was performed under dim green light. After spreading spores with a sterile cell spreader, plates were air dried for 15 min in a laminar flow hood.

To restrict directional illumination, plates were wrapped twice in black, light-absorptive aluminum foil, leaving only the upper edge of the plate exposed. Plates were positioned vertically in a climate chamber [Poly Klima Climatic Grow Systems, Hofmann Kühlung, firmware 03.10.10, controller WAGO 750-8212 PFC200 G2 2ETH R] at 23°C under continuous blue light (∼30 to 40 μmol/m^2^ per second; λ_max _= 450 nm) for 10 days.

For white light sporeling orientation assays (Fig. 3D), spores were sterilized and handled as described above, except that 100 μl of suspension was plated per square plate. Plates were incubated horizontally under continuous white light at 23°C. Images were acquired after 11 days using a Keyence microscope VHX-7000 as detailed above.

### miniTurbo-based proximity labeling and mass spectrometry

For miniTurbo-based proximity labeling (*50*), transgenic Tak-1 *M. polymorpha* lines expressing Mpphot-miniTurbo-YFP (*pro*Mp*PHOT*:Mp*PHOT*-*miniTurbo*-*YFP*_line-1 and line-2) and a plasma membrane control (*pro*Mp*PHOT*:Mp*PIP2-miniTurbo*-*YFP*), as well as the Tak-1 wild-type background, were grown from gemmae on half-strength B5 Gamborg medium supplemented with 1% sucrose, 1% plant agar, and MES hydrate (0.5 g/liter) (pH 5.5). Plants were grown for 10 days under standard white light on plates covered with cellophane to facilitate rapid thallus harvesting.

#### Proximity labeling under standard continuous white light

Ten-day-old thalli were incubated in 100 μM biotin dissolved in liquid half-strength B5 Gamborg medium for 16 hours. To stop the biotinylation, plant material was strained and rinsed three times with 200 ml of ice cold water, patted dry with paper towels, and flash frozen in liquid nitrogen. The frozen plant material was ground into a fine powder by a mortar and aliquoted into technical triplicates of 500 μl of plant powder per sample. Proteins were extracted by adding 1 ml of extraction buffer [50 mM tris (pH 7.5), 150 mM NaCl, 0.1% SDS, 1% NP-40, 0.5% Na-deoxycholate, 1 mM EDTA, 1 mM EGTA, 1 mM dithiothreitol, 20 μM MG-132, 1× cOmplete, and 1 mM phenylmethylsulfonyl fluoride], incubating for 15 min on a rotor wheel at 4°C. To digest cell walls, 1 μl of Lysonase (Millipore) was added per sample, followed by another 15-min incubation at 4°C before sonication in a water bath at high settings for 4 × 30 s with 90-s breaks. After sonication, samples were centrifuged at 15,000*g* for 15 min at 4°C, and the supernatants were desalted using PD-10 columns (GE Healthcare) to remove free biotin. The eluate was collected in 5-ml LoBind tubes (Eppendorf) and mixed with 200 μl of Dynabeads MyOne Streptavidin C1 (Invitrogen) slurry that was activated according to the manual and prewashed with extraction buffer. Streptavidin beads were incubated with the extracts for 16 hours at 4°C on a rotating wheel. Dynabeads were separated on a magnetic rack in 1.5 ml LoBind tubes (Eppendorf) and washed 4× with 1.5 ml of cold equilibration buffer, followed by washes with 1× with cold wash buffer I (equilibration buffer with 250 mM NaCl +1% SDS), 1× with cold equilibration buffer, 2× with cold wash buffer II (equilibration buffer with 250 mM NaCl), 2× with cold wash buffer III (equilibration buffer with 500 mM NaCl), 1× with cold equilibration buffer, and 2× with urea wash buffer [2 M urea and 50 mM tris (pH 7.5) at room temperature), as also described in Mair *et al.* (*51*) and Wallner *et al.* (*52*). Beads were resuspended in 1 ml of cold 50 mM tris (pH 7.5) and transferred into a new 1.5-ml LoBind tube, and all buffer was removed using a magnetic rack. Beads were frozen at −70°C for further processing.

#### Proximity labeling under dark and blue light conditions

The same Mpphot transgenic lines were grown and adapted in complete darkness for 24 hours. Plants were divided into three conditions: (i) labeling in darkness, (ii) blue light activation immediately upon transfer, and (iii) continuous blue light exposure for 4 hours before labeling. Biotin treatment was performed with 200 μM dissolved in liquid half-strength B5 Gamborg medium for 4 hours. Further processing of plant material was done as described for white light conditions.

#### On-bead digestion

Beads were resuspended in 60 μl of 100 mM ammonium bicarbonate with 600 ng of Lys-C (Fujifilm Wako) and incubated for 4 hours at 37°C on a thermoshaker (1200 rpm). The supernatant was reduced with 10 mM TCEP for 30 min at 60°C and alkylated with 40 mM MMTS for 30 min at room temperature. Digestion was completed with 600 ng of trypsin (Trypsin Gold, Promega) overnight at 37°C. Digests were acidified with 10 μl of 10% trifluoroacetic acid and submitted to liquid chromatography–tandem mass spectrometry (LC-MS/MS).

#### Nano–LC-MS/MS analysis

Peptides were separated on a Vanquish Neo UHPLC system coupled to an Orbitrap Exploris 480 mass spectrometer with FAIMS pro interface and Nanospray Flex source. Trap and analytical columns were PepMap Acclaim C18 (5 μm, 100 Å) with dimensions 5 mm–by–300 μm inner diameter (ID) (trap) and 500 mm–by–75 μm ID (analytical). Peptides were eluted at 230 nl/min using a 120-min linear gradient from 2 to 35% acetonitrile in 0.1% formic acid. Data were acquired in data-dependent acquisition mode with full scans at 60,000 resolution, a mass/charge ratio of 350 to 1200, and MS/MS scans of the most abundant ions with HCD at 30% collision energy. Compensation voltages of −45, −60, and −75 V were applied with cycle times of 0.9 s (CV, −45 and −60) and 0.7 s (CV, −75). Precursor ions of charge 2 to 6 were selected for fragmentation, with a dynamic exclusion of 45 s.

#### Data processing and analysis

RAW files were processed in Proteome Discoverer 2.5 with MSAmanda 2.0. Peptides were searched against the UniProt *M. polymorpha* database (34,956 sequences) and common contaminants. Fixed modifications were β-methylthiolation of cysteine, and variable modifications were methionine oxidation, phosphorylation (S/T/Y), deamidation (N/Q), N-terminal pyroglutamate, and N-terminal acetylation. FDR was set to 1% at PSM and protein levels using Percolator, and a minimum Amanda score of 150 was applied. Proteins were quantified with IMP-apQuant (MaxLFQ algorithm) and normalized using iBAQ with sum normalization across samples. Match between runs was applied, and proteins were filtered to require at least three quantified peptides per protein.

#### Volcano plot generation

Differential enrichment analysis was performed comparing each transgenic line to wild type and the control line. Volcano plots were generated in R studio using packages ggplot2 and ggrepel. Significantly enriched proteins were determined by *P* < 0.05 and a log_2_ fold change of >2.

#### Proximity labeling—Data availability

Mass spectrometry proteomics data from the proximity labeling experiments have been deposited to the ProteomeXchange Consortium via the PRIDE partner repository (*53*, *54*) with dataset identifier PXD072764.

### Quantification and statistical analysis

Sequence assemblies and plasmid maps were generated using CLC Genomics Workbench v9.5.1 (QIAGEN). Gene sequences were obtained from MarpolBase (https://marchantia.info/) (*48*). All experiments were repeated with three to five independent biological replicates. Sample sizes are indicated in the respective figure legends.

Data analysis and visualization were carried out using RStudio (v2022.07.2, Build 576) and GraphPad Prism (v8.1.1). Statistical analyses were performed in RStudio and GraphPad Prism.
