# Supplementary material for: Phototropin-mediated blue light signaling orients the asymmetry of Marchantia polymorpha spores
Source: Sci Adv. 2026 Nov 11;12(46):eaei3142. doi: 10.1126/sciadv.aei3142 (PMC13754033; doi:10.1126/sciadv.aei3142)
Supplement: Supplementary file 1 [file sciadv.aei3142_sm.pdf]

Science Advances
AAAS

Supplementary Materials for

Phototropin-mediated blue light signaling orients the asymmetry of *Marchantia polymorpha* spores

Johannes Roetzer *et al.*

Corresponding author: Liam Dolan, liam.dolan@gmi.oeaw.ac.at

Sci. Adv. **12**, eaei3142 (2026)
DOI: 10.1126/sciadv.aei3142

The PDF file includes:

Figs. S1 to S4
Legends for tables S1 and S2
Legends for data S1 and S2

Other Supplementary Material for this manuscript includes the following:

Tables S1 and S2

Figure S1

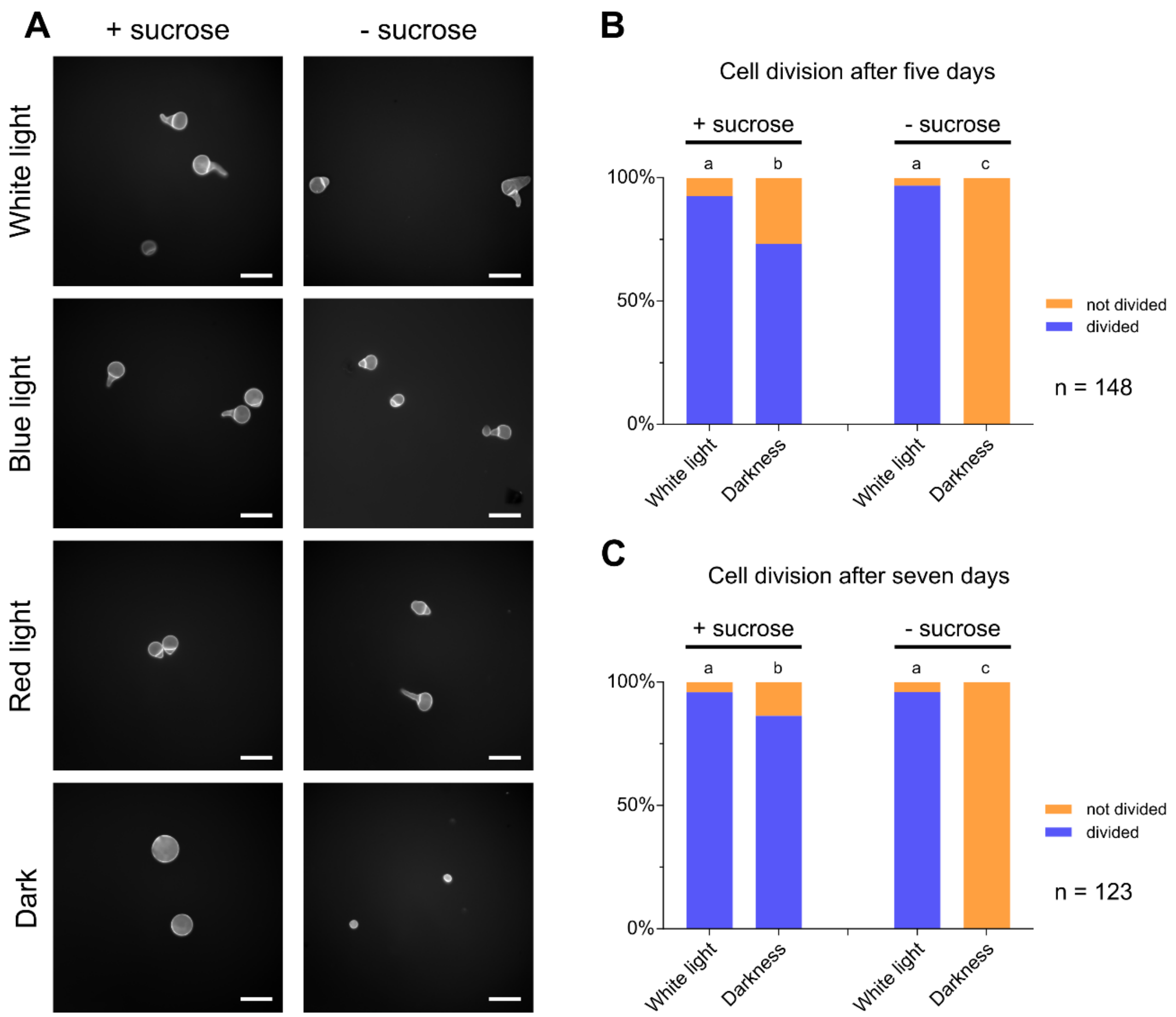

Fig. S1. Light positively regulates spore division in *M. polymorpha*. Related to Figure 1F-G. **(A)** Overview images of spores grown for 2 days in continuous darkness, white (PFD = 50-60 µmol m^{-2} s^{-1}), blue (PFD = 30-40 µmol m^{-2} s^{-1}, λmax = 450 nm), or red light (PFD = 80-90 µmol m^{-2} s^{-1}, λmax = 660 nm) on nutrient media with or without sucrose. Each overview image shows several spores used for quantification. Cell division occurred under all light conditions but not in darkness. Scale bars = 50 µm. **(B, C)** Quantification of spore cell division under continuous white light (PFD = 50-60 µmol m^{-2} s^{-1}) and darkness after 5 days (B; n = 148) and 7 days (C; n = 123), with and without sucrose. For both time points, light treatment had a significant effect on cell division with sucrose (5 days: $\chi^2(1) = 4.56$, $p = 0.033$; 7 days: $\chi^2(1) = 3.98$, $p = 0.046$; chi-square test) and without sucrose (5 and 7 days: Fisher's exact test, $p < 0.0001$). Different letters indicate statistically significant differences ($p < 0.05$); identical letters indicate no significant difference.

Figure S2

A

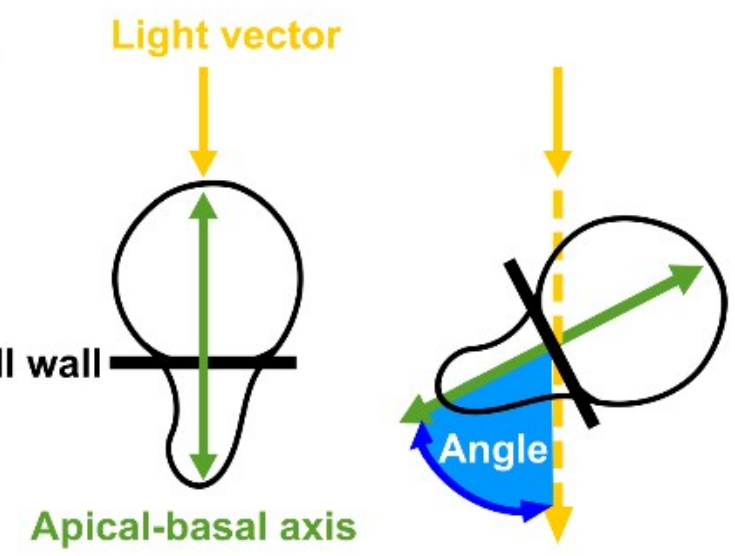

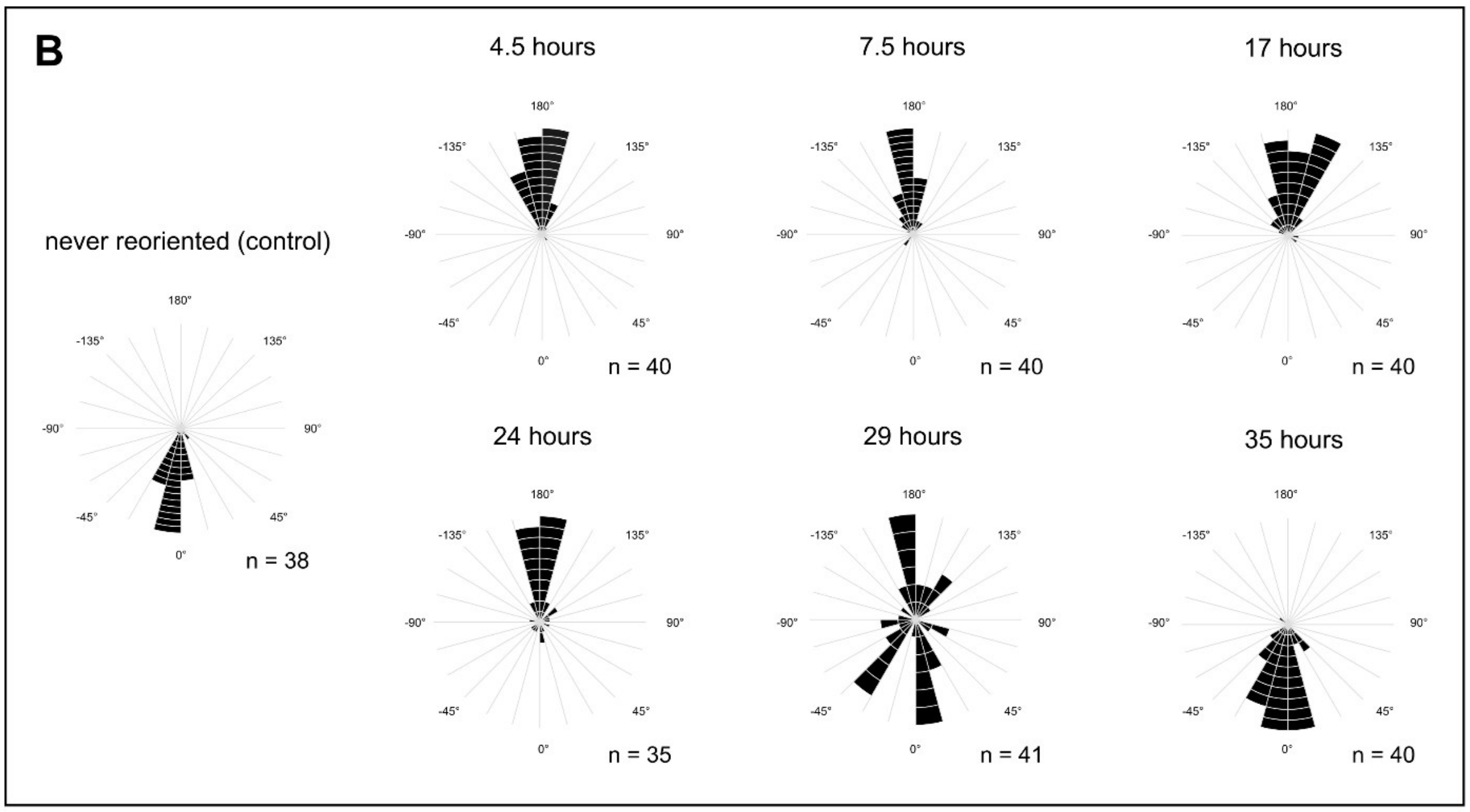

C

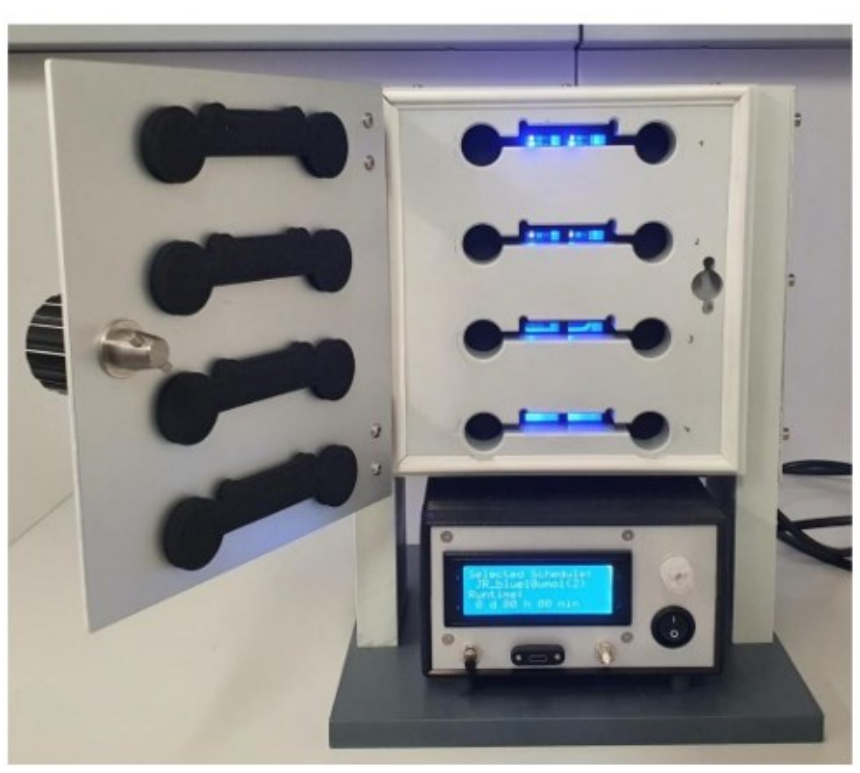

D

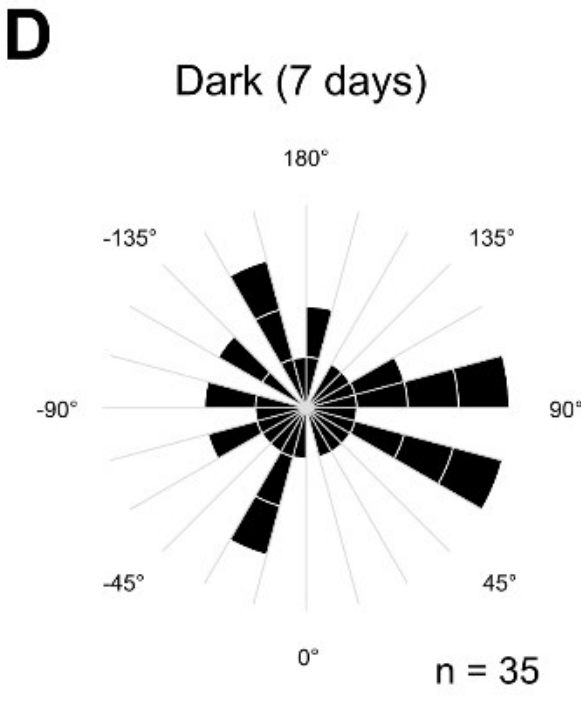

Fig. S2. Quantification and experimental setup for spore division orientation assays. Related to Figure 2.

(A) Schematic of cell division orientation measurement. When the new cell wall (black) is oriented perpendicular to the light vector (yellow), the apical–basal axis (green) aligns with the light vector (left). When the cell wall is not perpendicular to the light vector, the axes are misaligned (right). The angle (blue) between the light vector and the apical–basal axis was measured for all spores used in orientation assays. **(B)** Radial histograms of spores used for reorientation experiments (Figure 2C). Only asymmetrically dividing spores were included. Light direction was reversed at 4.5 h (n = 40), 7.5 h (n = 40), 17 h (n = 40), 24 h (n = 35), 29 h (n = 41), and 35 h (n = 40), with a non-reoriented control (n = 38). All spores were imaged and quantified after 48 h total illumination. **(C)** Image of the custom-built illumination system ("lightbox") used for spore orientation assays. The open-front view shows four units for chambered slides enabling unidirectional illumination. Details are described in *Materials and Methods*. **(D)** Radial histogram of spores dividing after 7 days in darkness on sucrose-supplemented medium (n = 35). Cell wall orientation was random.

Figure S3

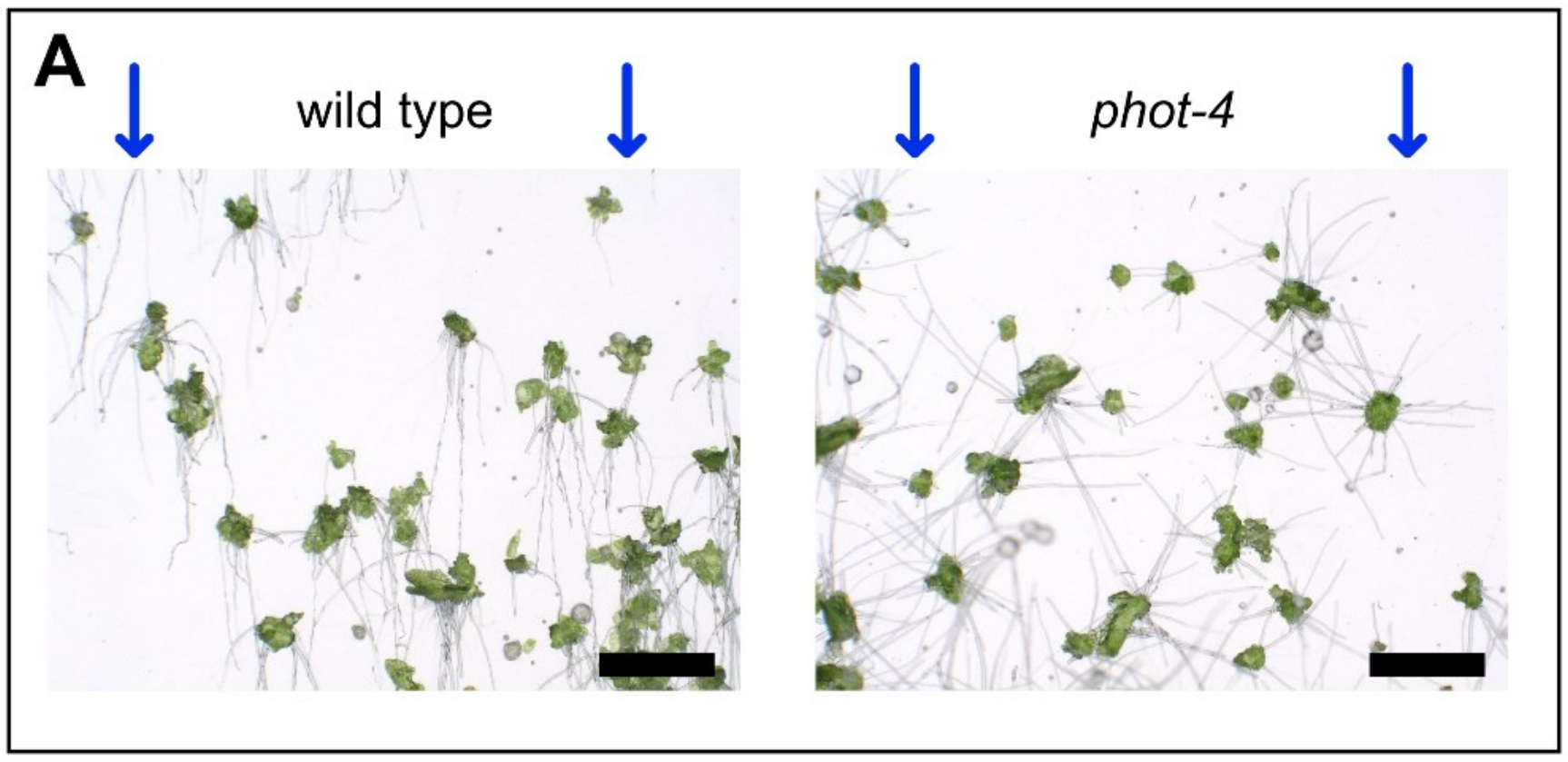

Fig. S3. Rhizoids of Mp*phot-4* mutant sporelings are defective in negative phototropism in blue light. Related to Figure 3C.
(A) Rhizoids of wild-type sporelings grew away from the blue light source, while Mp*phot-4* mutant rhizoids grew in multiple directions. Sporelings were grown for 10 days in continuous blue light (PFD = 30-40 µmol m^{-2} s^{-1}, λmax = 450 nm) on vertical plates with illumination from above. Scale bar = 1 mm.

Figure S4

A

Identifier	Available data / predicted function	No. of mutant lines
Mp5g07060	Mp*NCH1*	11
Mp8g10870	*BLUS*-like protein	17
Mp4g17810	*LEA-like29*	5
Mp6g16080	Kinesin light chain, TPR domain	40
Mp3g08670	Serine/threonine kinase	5
Mp3g18020	Receptor-like kinase	3
Mp3g05350	Mp*RLK-Pelle_RLCK-II* receptor-like kinase	3
Mp3g16650	*CDC2*-related protein kinase	20
Mp4g20380	Mp*PP2C_E,* phosphatase	7
Mp5g14150	Mp*PP2C_F4*, phosphatase	1
Mp7g08290	*unknown*	25

B

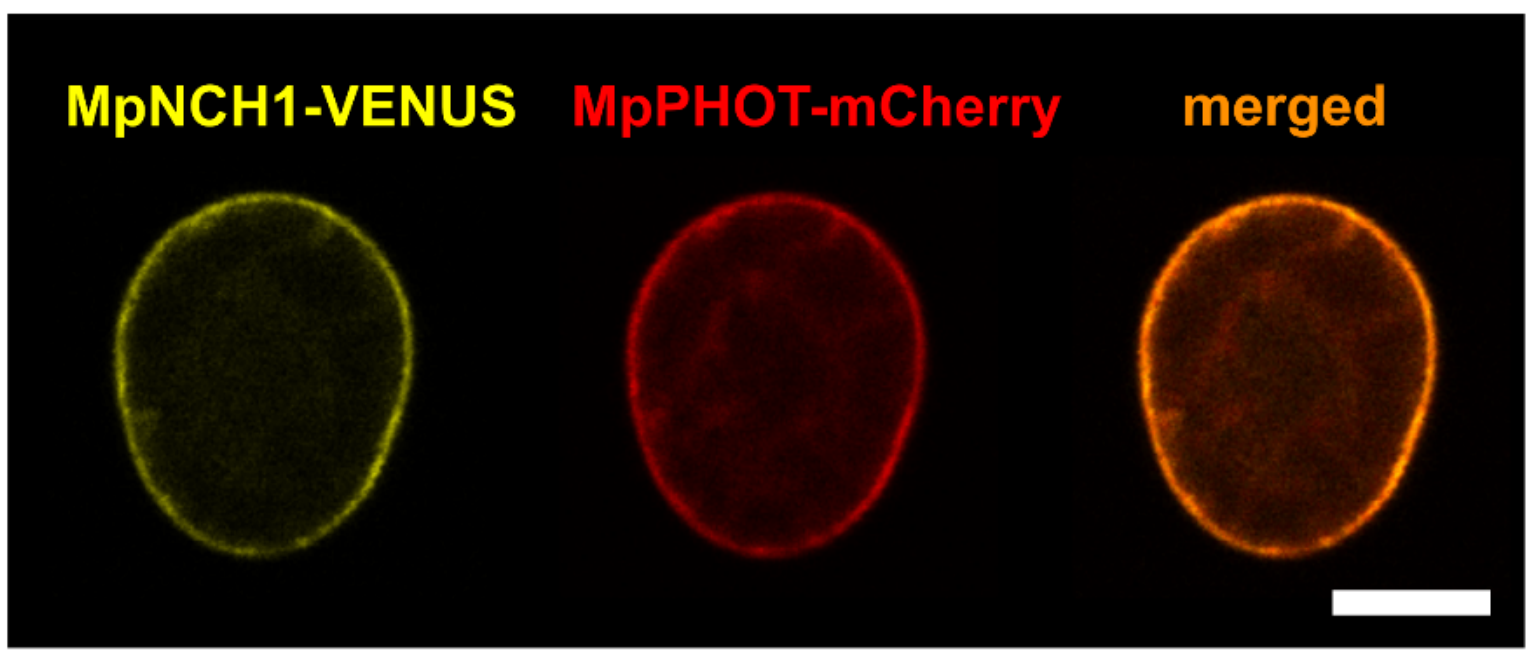

Fig. S4. Selection of candidate genes and co-localization of MpNCH1–VENUS and Mpphot–mCherry in the undivided spore. Related to Figure 4.
(A) A total of 137 loss-of-function mutant lines were generated for selected genes using CRISPR–Cas9. **(B)** MpNCH1–VENUS and Mpphot–mCherry co-localize in spores before the first cell division. Representative single-plane confocal images of an undivided spore are shown, including individual channels and the merged image. Co-localization analysis revealed a strong correlation between the signals (Pearson's r = 0.909), indicating overlapping subcellular localization. Spores were grown for 24 h under standard white-light conditions (PFD = 50-60 µmol m^{-2} s^{-1}) on cellophane-covered medium, then fixed, cleared, and imaged by confocal microscopy. Scale bar = 10 µm.

Other Supplementary Materials for this manuscript:

Table S1 (separate file). Summarized analysis of proximity labeling experiments. Related to Figure 4A.

Table S2 (separate file). Normalized read counts for all spore samples (RNA-seq). Related to Figure 3A.

Data S1 (available at https://zenodo.org/records/22947697). Schematic of the custom illumination system ("Lightbox") used for spore orientation assays.

Data S2 (available at https://zenodo.org/records/22947697). Schematic representations of the Mp*NCH1* and Mp*NPH3* genes and mutant alleles. Related to Figure 4.
sgRNA target sites and the predicted effects of each mutation on protein length are indicated.
